# Nonlinear response of soil microfauna network complexity and stability to multilevel warming in an old-growth subtropical forest

**DOI:** 10.1128/mbio.00156-25

**Published:** 2025-08-29

**Authors:** Debao Li, Yan Li, Haibian Xu, Jianping Wu

**Affiliations:** 1 State Key Laboratory for Vegetation Structure, Function and Construction, Ministry of Education Key Laboratory for Transboundary Ecosecurity of Southwest China, Yunnan Key Laboratory of Plant Reproductive Adaptation and Evolutionary Ecology and Institute of Biodiversity, School of Ecology and Environmental Science, Yunnan University 658481 , Kunming, Yunnan, China; 2 Laboratory of Soil Ecology and Health in Universities of Yunnan Province, Yunnan University 12635, Kunming, Yunnan, China; Oregon State University, Corvallis, Oregon, USA; The Ohio State University, Columbus, Ohio, USA

**Keywords:** multilevel warming, nonlinear response, network stability, network complexity, functional groups, multinutrient cycling

## Abstract

The influence of climate warming on soil microbes and the mechanisms underlying these effects have become the subject of intense focus in microbial ecology and climate change research. However, it is largely unknown how warming affects soil microfauna network complexity and stability or how warming-induced changes may affect ecosystem functioning in old-growth forests. Here, we conducted a 3-year multilevel warming experiment in an old-growth subtropical forest using infrared heating with five treatments: ambient soil temperature and 0.8°C, 1.5°C, 3.0°C, and 4.2°C above ambient soil temperature. We found that soil microfauna network complexity and stability and multinutrient cycling were significantly higher under warming and showed similar hump-shaped trends across rising temperatures. The nonlinear responses of soil microfauna network complexity and stability were primarily linked to soil temperature, moisture, organic carbon, and microbial biomass. Importantly, we found that soil multinutrient cycling was positively influenced by microfauna network complexity and stability. Consequently, our findings provide insights into the key role of soil microfauna network structure in regulating soil multinutrient cycling, highlighting the need to consider soil organisms’ potential interactions and that it is crucial to preserve soil microfauna “interactions” for ecosystem management in forests under global change.

**IMPORTANCE:**

It is largely unknown how warming affects soil microfauna network complexity and stability or how warming-induced changes may affect ecosystem functioning in old-growth forests. We conducted a 3-year multilevel warming experiment in an old-growth subtropical forest using infrared heating. We found that soil microfauna network complexity and stability were significantly higher under warming treatments and displayed nonlinear responses to different warming levels. Soil multinutrient cycling was positively and significantly influenced by microfauna network complexity and stability. Given that complex interconnections between soil microfauna are critical for sustaining ecosystem functioning, protecting microfauna “interactions” may be critical to mitigating the adverse impacts of warming-induced biodiversity reduction on ecosystem functioning.

## INTRODUCTION

Global mean air temperature continues to rise due to the rapid increase in atmospheric CO_2_ caused by human activities (1), with projections indicating arise of 1°C–4°C by 2100, and climate models predict that temperature variability will increase in the tropics and subtropics, accompanied by unprecedented warming (2). Global warming disrupts carbon and nitrogen exchanges between the atmosphere and Earth’s terrestrial ecosystems, but it remains unclear whether positive feedbacks driven by warming will increase soil greenhouse gas emissions (3), these impacts are highly dependent on soil organisms, which are key regulators of terrestrial carbon cycling due to their influence on carbon stabilization and mineralization, plant nutrient availability, and soil health (4). How soil organisms and their impact on ecosystem functioning are affected by global warming is thus crucial for predicting future climate regimes (3).

Soil microfauna primarily comprise nematodes and protists, are widespread in terrestrial ecosystems, and include various trophic levels in the soil food web, thereby influencing various ecological processes associated with human activities (5). Numerous studies have reported that warming affects soil nematode and protist diversity and community structure (6–9). However, populations of soil organisms do not exist in isolation but interact with one another to form complex communities and represent a critical dimension of community ecology (10). Ecological network analysis is a widely used and powerful tool for studying associations between organisms and how they are organized into communities (11). It also represents an approach for studying community complexity and stability and can be used to quantify how interactions between organisms contribute to ecosystem services and functions (10). Previous research demonstrates that the topological attributes of these networks can be altered by changing environmental conditions and that they can be used to assess ecosystems’ ability to respond to such changes (12, 13). Recent research has explored how climate warming influences bacterial and fungal network stability and complexity (12, 14). It is commonly recognized that ecosystem functioning is modulated by integrated soil organisms, instead of through single biota type (15). Soil microfauna, as relatively sedentary assemblages of organisms, are more vulnerable to environmental changes and therefore may act as beneficial bioindicators (16). Moreover, compared to changes at lower trophic levels, changes at higher trophic levels have comparably or even greater impacts on ecosystem functions (15). However, the responses of soil microfauna network complexity and stability to warming and consequently influence on ecosystem functions related to nutrient cycling remain unclear.

Nematodes form critical components of the soil food web because of their roles as omnivores, predators, herbivores, fungivores, and bacterivores (17). They help regulate nutrient and carbon dynamics, control soil microbial communities, and influence nutrient recycling and decomposition (17). Protist functional groups include consumers, parasites, and phototrophs, which regulate multiple underground ecological processes and affect the aboveground plant communities through interaction with the fungal and bacterial communities (18). Considering the significance of soil microfauna for nutrient cycling, mineralization, and decomposition, predicting how warming will influence microfauna functional groups is crucial for projecting changes in soil health and food web structure under climate change (19). Previous research suggested that warming affects the abundance of soil microfauna functional groups directly by impacting reproduction and development and indirectly through changes in food supply by altering microbial activity and plant organic inputs to soil (19). Nevertheless, it is unclear whether and how warming-induced alterations in soil microfauna functional groups influence soil multinutrient cycling.

The Sixth Assessment Report of the Intergovernmental Panel on Climate Change predicts future conditions under five different climate scenarios, called Socio-economic Pathways (SSPs) (1). The report predicts that global average surface air temperatures between 2081 and 2100 will exceed those of the recent past (1995–2014) by 0.7°C in the low-emissions scenario SSP1-1.9, 1.2°C in SSP1-2.6, 2.0°C in SSP2-4.5, 3.1°C in SSP3-7.0, and 4.0°C in the high-emission scenario SSP5-8.5 (1). In light of existing climate policies as well as past and current CO_2_ emissions, higher-emissions scenarios (SSP1-2.6, SSP2-4.5, SSP3-7.0, or SSP5-8.5) may be more aligned with our current trajectory than lower-emission scenarios (20). A better understanding of how soil microfauna respond to higher temperatures is critical for improving predictions of how these ecosystems will change under climate warming and for informing strategies to protect soil organisms. Here, we conducted a 3-year warming simulation in a primary subtropical forest with five warming levels: ambient temperature (control), ambient +0.8°C, ambient +1.5°C, ambient +3.0°C, and ambient +4.2°C. We explored two questions: (i) whether and how multilevel warming influences soil microfauna network complexity and stability, as well as the relative abundance of soil microfauna functional groups and (ii) whether and how multilevel warming-induced alterations of soil microfauna network complexity and stability influence soil multinutrient cycling.

## RESULTS

### Soil microfauna network stability and complexity

**Fig 1:**
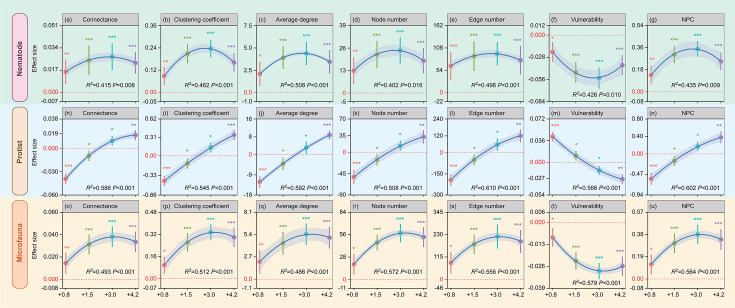
Effect sizes of multilevel warming on soil nematode (a–g), protist (h–n), and whole microfauna (o–u) network complexity and stability indices in the 0–10 cm soil layer as obtained from linear mixed-effects models. Results are expressed as mean ± standard error of the estimated effect sizes. Statistical significance is based on Wald type II χ² tests; ***P < 0.001, **P < 0.01, *P < 0.05. Regression lines are blue and gray shading denotes 95% confidence intervals. +0.8°C, 0.8°C above ambient temperature; +1.5°C, 1.5°C above ambient temperature; +3.0°C, 3.0°C above ambient temperature; +4.2°C, 4.2°C above ambient temperature; NPC, |negative|:positive cohesion.

Soil nematode and whole microfauna network complexity and stability showed hump-shaped trends with temperature increase in the topsoil (Fig. 1a through g and o through u). Increases in soil nematode complexity and stability were largest in the +3°C treatment, followed by increases under +1.5°C and +4.2°C treatments (Fig. 1a through g). Increases in soil nematode network complexity and stability were smallest in the +0.8°C treatment (Fig. 1a through g). In addition, increases in soil whole microfauna network complexity and stability were largest in the +3°C treatment, followed by increases under +4.2°C and +1.5°C treatments (Fig. 1o through u). Increases in soil whole microfauna network complexity and stability were smallest in the +0.8°C treatment (Fig. 1o through u).

Soil protist network complexity and stability were significantly lower in the topsoil under +0.8°C and +1.5°C treatments but significantly higher in the +3°C and +4.2°C treatments (*P* < 0.05) (Fig. 1h through n). Declines in soil protist network complexity and stability were largest in the +0.8°C treatment (Fig. 1h through n) and increases were greatest under +4.2°C treatment (Fig. 1h through n). Warming did not significantly influence soil nematode and protist, and whole microfauna network complexity and stability in the subsoil (Fig. S3).

### Mechanisms underlying altered soil microfauna network complexity and stability

**Fig 2:**
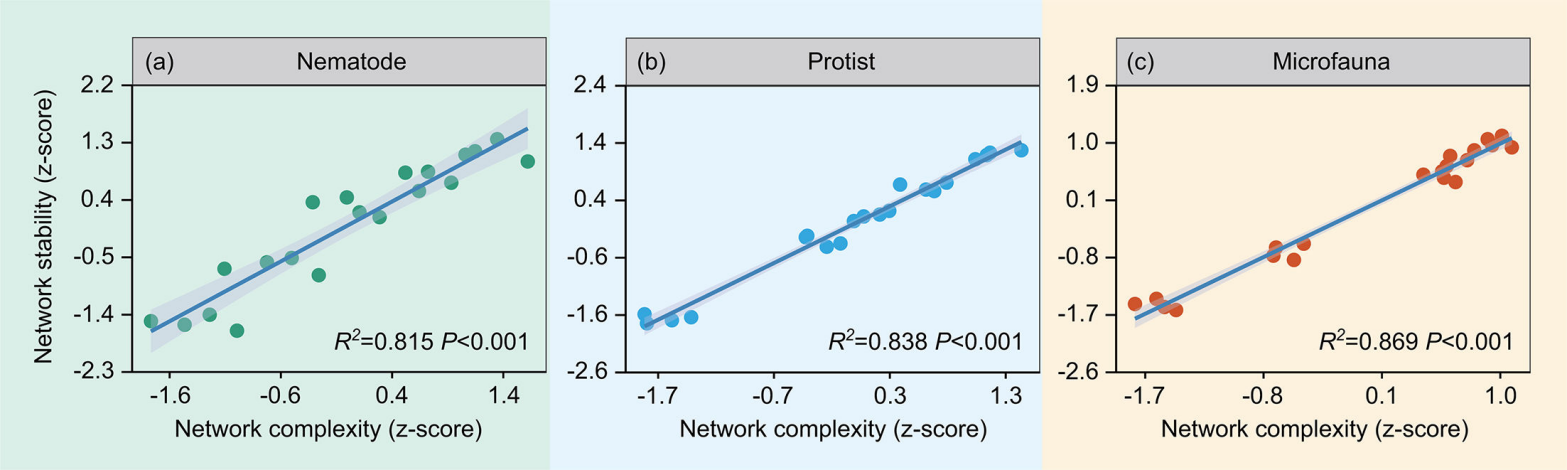
Links between soil nematode network stability and nematode network complexity (a) in the 0–10 cm soil layer. Links between soil protist network stability and protist network complexity (b) in the 0–10 cm soil layer. Links between soil whole microfauna network stability and whole microfauna network complexity (c) in the 0–10 cm soil layer. Regression lines are blue and gray shading denotes 95% confidence intervals.

Regression analyses showed that soil nematode and whole microfauna network complexity and stability had significant unimodal correlation with soil moisture, temperature, and organic carbon and linear and negative correlation with soil microbial biomass phosphorus in the topsoil (*P* < 0.001) (Fig. S4 and S5). Nematode and whole microfauna network stability and complexity were not significantly correlated with other soil properties (Fig. S6 to S9). Soil protist network stability and complexity were linearly and negatively correlated with soil-dissolved organic carbon, microbial biomass nitrogen and carbon, pH, total phosphorus, total nitrogen, and NH_4_^+^-N in the topsoil (*P* < 0.001) (Fig. S4, S5, S10, and S11). Furthermore, we found that soil nematode, protist, and whole microfauna network stability were linearly and positively correlated with network complexity, respectively (*P* < 0.001) (Fig. 2).

**Fig 3:**
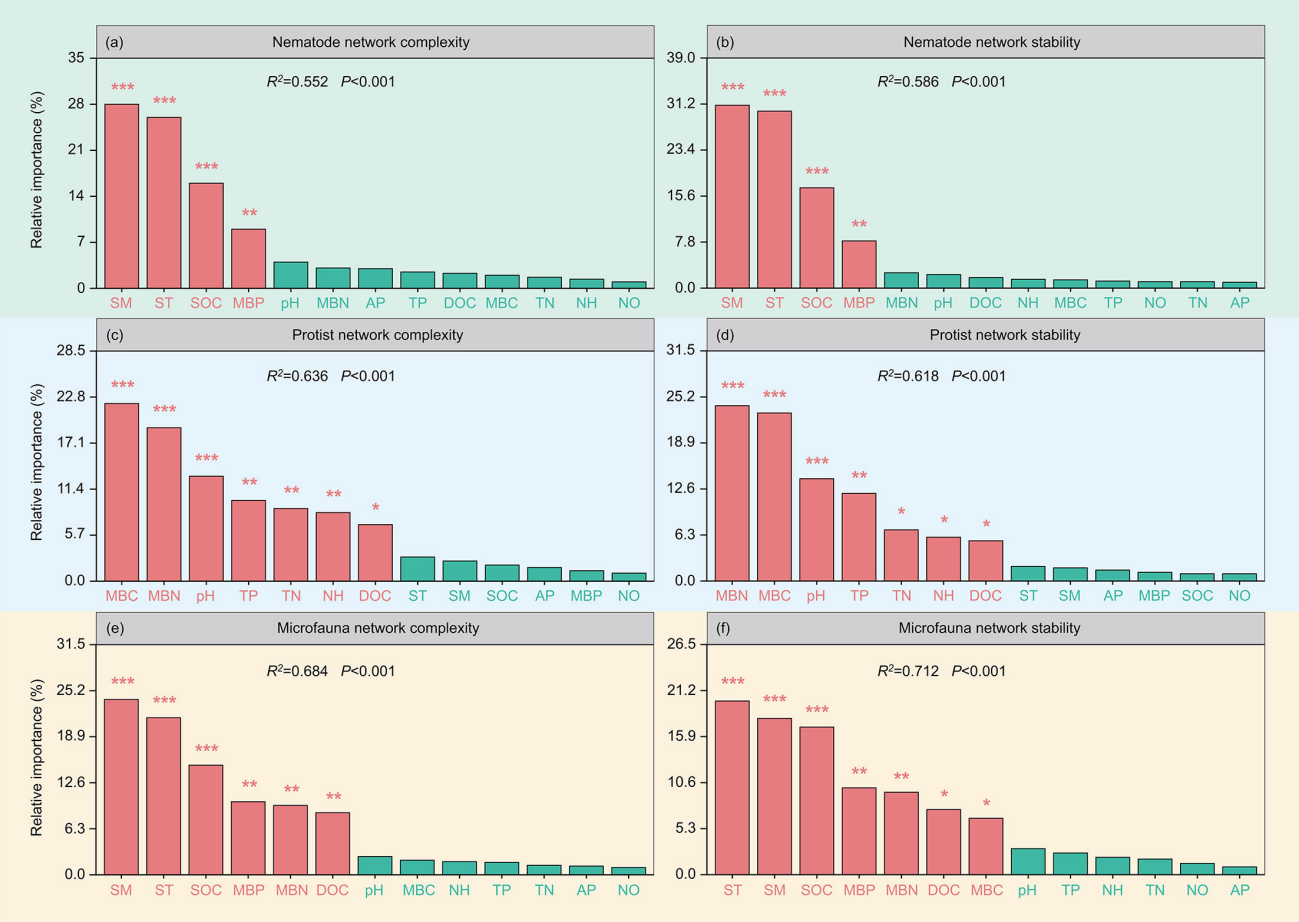
Relative importance of environmental factors driving variation in soil nematode network complexity (a) and stability (b), protist network complexity (c) and stability (d), and whole microfauna network complexity (e) and stability (f) based on hierarchical variance partitioning in the 0–10 cm soil layer. *P* values were calculated from linear mixed-effects models, *P < 0.05, **P < 0.01, ***P < 0.001. SOC, soil organic carbon; SM, soil moisture; ST, soil temperature; AP, soil available phosphorus; MBN, soil microbial biomass nitrogen; MBP, soil microbial biomass phosphorus; pH, soil pH; TP, soil total phosphorus; NO, soil NO_3_^-^-N; NH, soil NH_4_^+^-N; TN, soil total nitrogen; MBC, soil microbial biomass carbon; DOC, soil dissolved organic carbon.

Soil moisture and temperature were the dominant drivers of changes in soil nematode and whole microfauna network stability and complexity in the topsoil (*P* < 0.001) (Fig. 3a and b, e and f). In addition, soil nematode network complexity and stability were significantly influenced by soil microbial biomass phosphorus and organic carbon (*P* < 0.01) (Fig. 3a and b). Soil whole microfauna network complexity and stability were significantly influenced by soil organic carbon, microbial biomass phosphorus and nitrogen, and dissolved organic carbon (*P* < 0.05) (Fig. 3e and f). Soil microbial biomass carbon and nitrogen had the greatest impact on protist network complexity and stability (*P* < 0.001) (Fig. 3c and d). Moreover, soil pH, dissolved organic carbon, total nitrogen, total phosphorus, and NH_4_^+^-N significantly affected soil protist network complexity and stability (*P* < 0.05) (Fig. 3c and d).

### Links between soil multinutrient cycling and microfauna network structure

**Fig 4:**
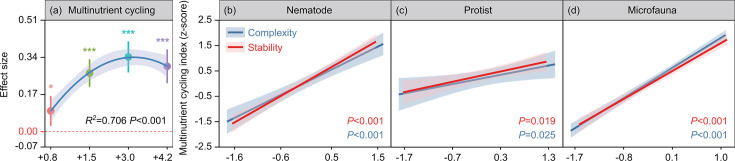
Effect sizes of multilevel warming on multinutrient cycling (a) in the 0–10 cm soil layer as obtained from linear mixed-effects models. Results are expressed as mean ± standard error of the estimated effect sizes. Statistical significance is based on Wald type II χ² tests; ***P < 0.001, **P < 0.01, *P < 0.05. Regression lines are blue and gray shading denotes 95% confidence intervals. Links between soil nematode (b), protist (c), and whole microfauna (d) network complexity and stability and multinutrient cycling in the 0–10 cm soil layer, as obtained from generalized linear mixed-effects models. Lines and shaded polygons indicate generalized linear mixed-effects model predictions and their 95% confidence intervals. +0.8°C, 0.8°C above ambient temperature; +1.5°C, 1.5°C above ambient temperature; +3.0°C, 3.0°C above ambient temperature; +4.2°C, 4.2°C above ambient temperature.

Soil multinutrient cycling followed a hump-shaped association with the temperature increase, with the greatest increase in the +3°C treatment, followed by increases under +4.2°C and +1.5°C treatments in the topsoil (Fig. 4a). Increases in soil multinutrient cycling were smallest in the +0.8°C treatment (Fig. 4a). Soil nematode, protist, and whole microfauna network stability and complexity significantly and positively impacted multinutrient cycling (Fig. 4b through d). In addition, soil multinutrient cycling had a significant unimodal correlation with soil moisture and temperature (Fig. S12). Warming did not significantly change soil multinutrient cycling in the subsoil (Fig. S13).

### Soil microfauna functional groups

**Fig 5:**
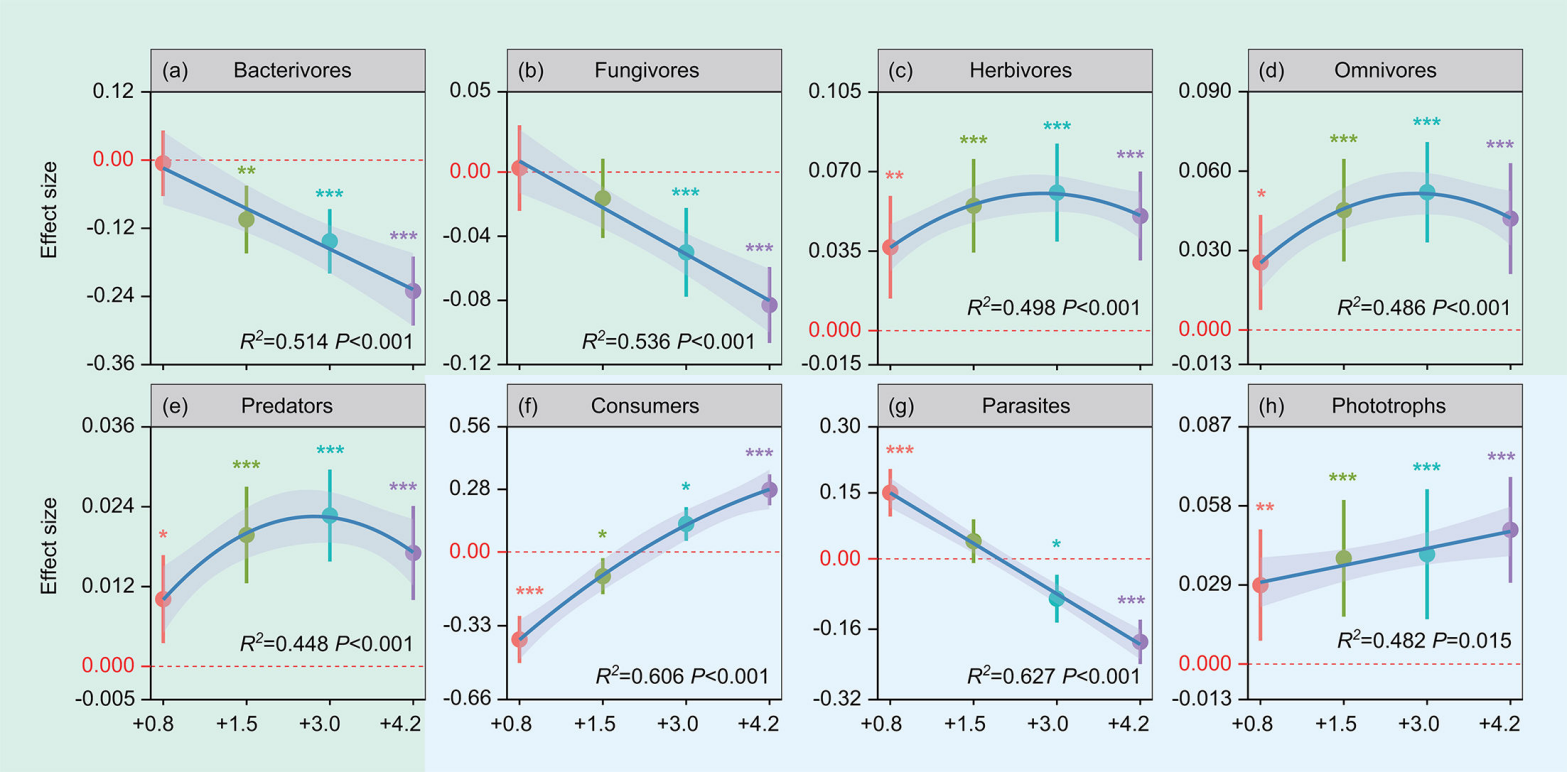
Effect sizes of multilevel warming on the relative abundance of nematode functional groups (a–e) and protist functional groups (f–h) in the 0–10 cm soil layer as obtained from linear mixed-effects models. Results are expressed as mean ± standard error of the estimated effect sizes. Statistical significance is based on Wald type II χ² tests; ***P < 0.001, **P < 0.01, *P < 0.05. Regression lines are blue and gray shading denotes 95% confidence intervals. +0.8°C, 0.8°C above ambient temperature; +1.5°C, 1.5°C above ambient temperature; +3.0°C, 3.0°C above ambient temperature; +4.2°C, 4.2°C above ambient temperature.

**Fig 6:**
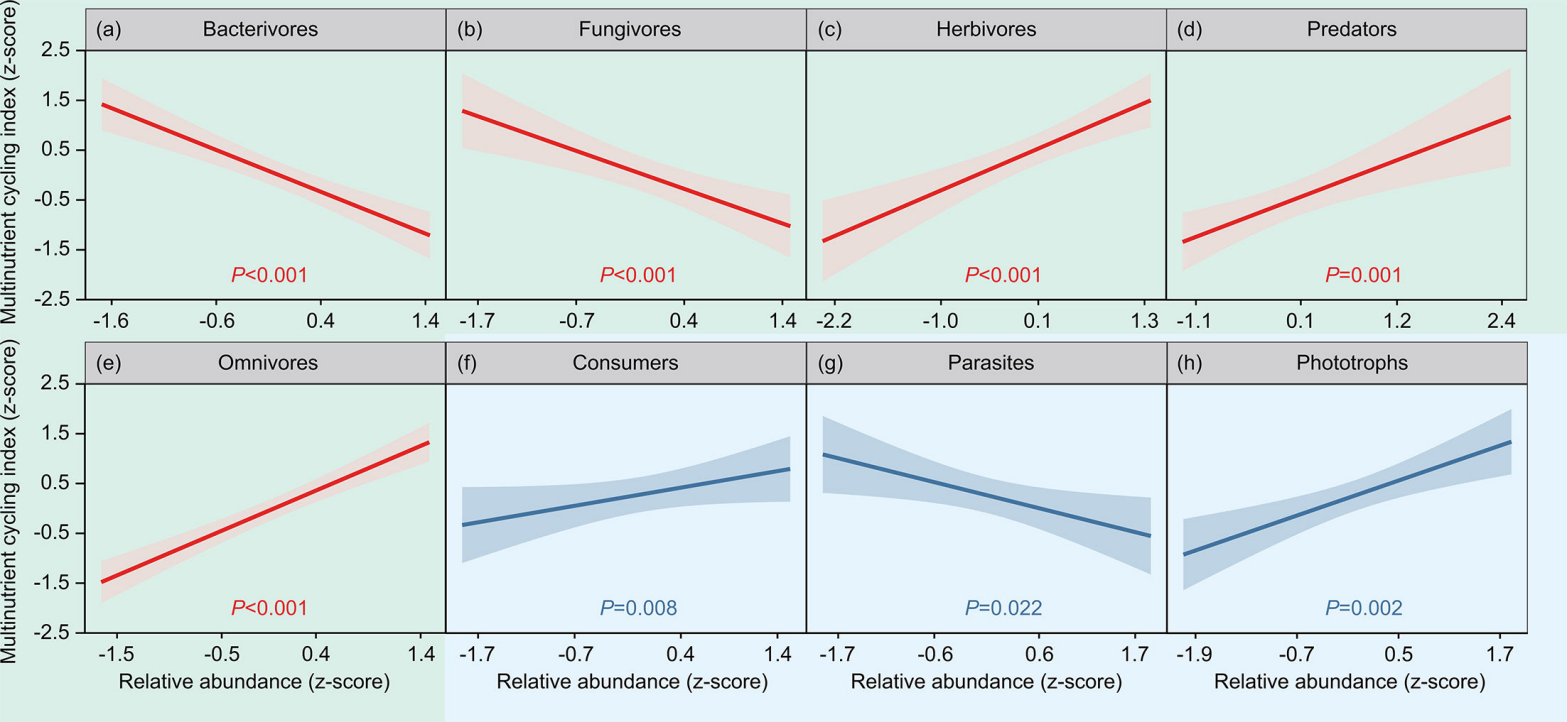
Links between the relative abundance of nematode functional groups (a–e) and protist functional groups (f–h) and multinutrient cycling in the 0–10 cm soil layer, as obtained from generalized linear mixed-effects models. Lines and shaded polygons indicate generalized linear mixed-effects models predictions and their 95% confidence intervals.

The relative abundance of bacterivores was not significantly altered under the +0.8°C treatment but was significantly lower under +1.5°C, +3.0°C, and +4.2°C treatments in the topsoil (*P* < 0.01) (Fig. 5a). +0.8°C and +1.5°C treatments had no significant influence on the relative abundance of fungivores, but +3.0°C and +4.2°C treatments decreased it (*P* < 0.001) (Fig. 5b). All warming treatments significantly and positively influenced the relative abundance of herbivores, predators, and omnivores (*P* < 0.05) (Fig. 5c through e). The relative abundance of protist consumers decreased significantly under +0.8°C and +1.5°C treatments but increased significantly in the +3°C and +4.2°C treatments (*P* < 0.05) (Fig. 5f). Under 0.8°C treatment, the relative abundance of protist parasites increased significantly (*P* < 0.001) while there was no change in the +1.5°C treatment. The relative abundance of protist parasites decreased significantly under +3°C and +4.2°C treatments (*P* < 0.05) (Fig. 5g). All warming treatments significantly enhanced the relative abundance of protist phototrophs (*P* < 0.01) (Fig. 5h). Furthermore, generalized linear mixed-effects model results showed that soil microfauna functional groups significantly influenced multinutrient cycling (*P* < 0.05) (Fig. 6). Warming did not alter soil microfauna functional groups significantly in the subsoil (Fig. S14).

## DISCUSSION

### Nonlinear response of soil microfauna network stability and complexity to multilevel warming

Environmental filtering drives species co-occurrence patterns (21) and influences soil organism network stability and complexity (12). Soil temperature and moisture change simultaneously due to warming, with soil temperature and moisture thought to be tightly associated with soil organisms’ metabolic rates and biochemical processes (22). Moisture and temperature may thus directly constrain network community formation by influencing the metabolic activity of soil organisms and by influencing their evolution and processes related to community assembly (22). Normally, nematodes are active at temperatures between 5°C and 30°C, with an optimum temperature of around 20°C (23). Warming likely increased nematode network complexity and stability by shifting temperatures toward this optimum at the study site, where the mean annual temperature is 11.11°C. Soil moisture also regulates nematode survival, development, and movement (24), and alterations in soil moisture can change nematode community structure, influencing network complexity and stability (25). Reducing aeration in saturated soils is commonly harmful to nematodes, and it has been demonstrated that when soil moisture varies from saturated to slightly drier conditions, the fastest rate of development and movement of many nematodes occurs (23). Soil moisture is high in humid subtropical areas (Fig. S2), which are characterized by frequent rainfall and high mean annual precipitation (14). The moderate warming (+3.0°C) in our study may have caused saturated soil to slightly dry out, increasing the rate at which soil nematodes develop and move, thereby increasing network complexity and stability. Nevertheless, nematode actions, including reproduction and feeding, require sufficient soil moisture (26). Intense warming (+4.2°C) enhances soil transpiration, exacerbating thermal stress and soil drying. Thus, the most intense warming treatment may have irreversibly altered nematodes’ habitats, thereby decreasing network complexity and stability (26). Although small and moderate increases in temperature can increase soil nematode network stability and complexity, the declines in soil moisture induced by larger temperature increases may mitigate this positive effect. These findings suggest that nonlinear variations in soil nematode network complexity and stability can result from the combined influence of higher soil temperature and lower moisture.

Soil organic carbon decomposition is highly temperature dependent, and warming can enhance its rate of decomposition, leading to soil organic carbon reduction (27). In addition, several studies have demonstrated that warming can encourage plants to take up large amounts of phosphorus, putting microbes at a disadvantage in the phosphorus competition (28–30). The quantity of microbial fixed phosphorus was reduced, resulting in a decrease in microbial biomass phosphorus (28). The findings suggest that warming tends to inhibit soil microbial biomass phosphorus, thus causing nutrient limitation in microfauna (31). Although species interactions are inherently self-interested, reductions in carbon availability and nutrient limitation impose constraints on cellular proliferation, necessitating improvements in stress tolerance and resource acquisitions within the soil organism community (32). Corresponding strategies include symbiosis-induced functional complementation, by-product cross-feeding, and horizontal gene transfer hinging on interspecific interactions, particularly at inter-trophic levels (33). This can lead to increasing network complexity and stability with declining soil organic carbon and nutrient limitation (33).

Consistent with previous findings (13), soil protist network complexity and stability were linearly and negatively linked to microbial biomass nitrogen and carbon. Previous work has shown that microbial biomass nitrogen and carbon increase under low-level warming but decrease as warming intensifies (34). Warming-induced increases in nitrogen availability and fresh carbon input from below- and above-ground plant parts could enhance microbial growth and be activated under low and medium levels of warming, resulting in increases in soil microbial biomass nitrogen and carbon (34, 35). However, lower soil moisture under elevated temperatures can inhibit microbial growth and activity, potentially countering those increases and reducing microbial biomass nitrogen and carbon under high-level warming (36). Soil protists are key microbial predators, and changes in microbial biomass caused by warming can affect the selective feeding patterns of various protist taxa, thus changing protist network complexity and stability (13, 37). High soil microbial biomass reduced soil protist network stability and complexity, but low microbial biomass increased it, supporting the stress gradient hypothesis (38). Previous research suggests that stressful conditions promote metabolic interactions between soil organisms, increasing interconnections within cross-feeding networks (39). Such stronger associations can thus increase network stability by improving soil organisms’ resistance to environmental stress (13).

Soil microfauna network stability was positively and significantly linked to network complexity in the topsoil (Fig. 2). More complex networks are generally more stable, indicating that higher interspecific interactions can promote community stability (40). Our findings are consistent with the hypothesis that ecosystem stability increases with complexity (41). Recent studies have also emphasized that changes in network stability with environmental stresses or warming could be primarily caused by species interaction processes since relative abundances of different taxa altered significantly correspondingly (12, 42).

### Differential responses of soil microfauna functional groups to multilevel warming

Previous works have revealed that low warming levels do not affect, and high warming levels significantly decrease the relative abundance of bacterivores and fungivores (43, 44), which is similar to our results. Many studies have reported that low-level warming does not significantly influence soil bacterial and fungal diversity, whereas high-level warming significantly reduces their diversity (45, 46). Furthermore, warming-induced reductions in soil moisture levels reduce cellular water potential and the mobility of soluble substrates, both of which can alter microbial diversity (47). Higher temperature causes greater soil moisture loss, thereby intensifying the warming impact on microbial diversity. Reduction of soil microbial diversity thereby leading to a decrease in the relative abundance of fungivores and bacterivores (47). All warming treatments significantly enhanced the relative abundance of herbivores, predators, and omnivores (Fig. 5c through e). This finding was consistent with our expectations since warming-induced increases in belowground plant productivity increase these nematodes’ food supply (48). High relative abundances of predators and omnivores suggest that the system is complex and resistant, with a degree of natural tolerance for disturbance (49).

Our findings are consistent with previous reports that warming feedbacks differ among protist functional groups (6). This may be because the optimal temperature for development and growth differs between some taxa (9). For instance, previous work found that heterotrophic flagellates are dominant in the soil at 5°C, while amoeboid protists are dominant at 23°C (50). Furthermore, warming could influence protist functional groups by changing soil moisture. Soil protists require moisture to be active, so their functions are strongly constrained by the availability of moisture in soil pore spaces (51). Protists with different body sizes and lifestyles can tolerate different degrees of soil dryness, with larger taxa generally having greater effects from aridity than smaller taxa (52). For instance, the diversity of heterotrophic protists is generally highest in continuously wet soils (52), whereas some species (e.g., dictyostelid cellular slime molds) exhibit greater diversity in soils that alternate between dry and wet conditions (51).

### Soil microfauna network stability and complexity influence multinutrient cycling

Relationships between microfauna network complexity and stability and soil multinutrient cycling were positive, which is supported by previous studies (53, 54). Following the explanations may contribute to this result. First, a more complex and stable soil microfauna network can comprise more predators that prey on fungi and bacteria, therefore promoting the availabilities of soil nutrients and carbon through the release of nutrients trapped in microbial biomass (13). Second, a more complex and stable network offers greater benefits in terms of information transfer and resources used to underpin various functions (55). Third, interaction between predators and prey in soil microfauna networks can improve functional synergies of microbes through the selective promotion of metabolically active microbes or alterations in microbial community composition (15). Warming-induced alterations in the relative abundance of soil microfauna functional groups can regulate network stability and complexity via top-down effects, thus influencing its mediated ecosystem functioning (56). Furthermore, soil multinutrient cycling can be directly and indirectly influenced by abiotic drivers by changing soil microfauna network stability and complexity. For instance, soil moisture and temperature can directly affect soil multinutrient cycling by influencing consumer, predator, and decomposer activities and interactions, and can indirectly influence soil multinutrient cycling via altering network stability and complexity (15). Complex and stable networks support diverse interactions and functional complementarity (57), whereas network simplicity can increase functional homogenization, lowering organisms’ stress tolerance (58). Consequently, maintaining diverse interactions may be key to mitigating the adverse impacts of warming-induced biodiversity decline on ecosystem functioning (12).

### Effects of multilevel warming vary with the soil layer

Numerous studies have demonstrated that the microfauna community structure varies between soil layers, consistent with our findings (59, 60). Here, the influence of multilevel warming on microfauna network complexity and stability and functional groups varied with soil layer, suggesting that soil microfauna response to climate warming is spatially heterogeneous across depths because of the alteration of both abiotic and biotic factors. Our finding that multilevel warming had significant effects on network stability and complexity and functional groups only in the topsoil (0–10 cm) is consistent with the hypothesis that soil organism processes are controlled by soil organic carbon quality and quantity and soil nutrient status (61). Differences between soil layers likely result from greater availability of labile carbon in the topsoil, which may be more vulnerable to environmental variability than the recalcitrant carbon that accumulates in the subsoil (10–20 cm) (62). Furthermore, the amount of substrate available to support the activity and metabolism of soil organisms is relatively adequate in topsoil because of the input of fresh carbon from roots and litter (60). In addition, topsoil temperature and moisture changes caused by experiment treatments are more rapid and severe than those in the subsoil. As a result, soil nutrients are less variable in the subsoil (60).

We recognize that robust traditional morphology-based approaches for investigating soil microfauna are limited in our study. A cumulative investigation of the diversity of soil microfauna may not be allowed due to soil sample volume and primer biases. But there is no comprehensive perspective from any single approach. For example, morphological examination for soil microfauna taxa determination is still a task that needs expertise and considerable time, with intrinsic potential for biases. To comprehensively characterize soil microfauna community structure and trophic traits, it is ideal to use a combination of traditional morphology-based approaches and high-throughput sequencing techniques (63).

### Conclusions

Our findings have several important implications for predicting ecological consequences and ecosystem management for future climate warming in old-growth subtropical forests. First, climate warming stimulated dynamic responses that caused higher soil microfauna network stability and complexity, which make the ecosystem potentially less vulnerable to a warmer world. Second, soil microfauna network stability and complexity are positively related to multinutrient cycling. Therefore, protecting network structure is essential for future ecosystem preservation. Finally, because of accelerating dynamic responses, biodiversity may be altered more rapidly under future warming scenarios, potentially causing biodiversity to decline. As a result, its associated ecosystem services and functions can become more vulnerable. Given that complex interconnections between soil microfauna are critical for sustaining ecosystem functioning, protecting microfauna “interactions” may be critical to mitigating the adverse impacts of warming-induced biodiversity decline on ecosystem functioning. In the context of global change, these soil organism co-occurrence networks should be highlighted and incorporated into ecosystem management.

## MATERIALS AND METHODS

### Study location

This experiment was set up in a subtropical evergreen broadleaf forest at the Ailaoshan Station for Subtropical Forest Ecosystem Studies on Ailao Mountain, Jingdong County, Yunnan Province, China (101° 01′E, 24°32′N; 2480 m elevation). This type of forest is well protected and widely distributed across this area, where the average stand age is >300 years. Dominant species include *Pteridium aquilinum* var. *latiusclum*, *Carex teinogyna*, *Sinarundinaria nitida*, *Castanopsis rufescens*, *Lithocarpus hancei*, and *Lithocarpus xylocarpus* (45). Mean annual precipitation and temperature are 1883.98 mm and 11.11°C, respectively, and were calculated using data collected from 1982 to 2021 (47). Soils in this area are classified as loamy Alfisols (45).

### Experimental design

The study was initiated in May 2019 and employed a random block design (Fig. S1). Briefly, we established four experimental blocks divided into five 2 × 2 m^2^ sections for a total of 20 plots. Plots were warmed using an infrared heating system, with each of the five plots randomly assigned one of five treatments: ambient temperature (control), ambient +0.8°C, ambient +1.5°C, ambient +3.0°C, and ambient +4.2°C (Fig. S2). A detailed description of this experimental design was presented in the supplemental material.

### Soil sampling and measurements

In August 2022, five 3 cm diameter soil cores were collected from the topsoil (0–10 cm) and subsoil (10–20 cm) in randomly selected locations within each plot, after which soil was mixed to form a single composite sample for each plot. Soil samples were stored in a cooler on ice and transported to the laboratory, where they were passed through a 2 mm sieve to exclude stones, litter, and roots. Samples were then split into two subsamples: one was stored at 4°C to measure soil properties. The second subsample was stored at −80°C for molecular analyses to profile microfauna communities. Procedures used for soil physicochemical property analyses, DNA extraction, Illumina sequencing, and bioinformatics analyses are detailed in the Supplementary Information.

### Statistical analyses

Co-occurrence networks for nematodes, protists, and the two above communities were constructed independently to examine potential links among members in topsoil and subsoil, respectively (i.e., a total of 6 networks were constructed, each containing 20 samples). Co-occurrence networks were generated using Spearman’s correlation matrices with the WGCNA package. Prior to network construction, we used random matrix theory (RMT) to automatically select appropriate Spearman correlation thresholds (64). A series of appropriate thresholds were determined for nematode (0.82), protist (0.84), and whole microfauna (0.87) networks in topsoil and for nematode (0.76), protist (0.79), and whole microfauna (0.84) networks in subsoil based on random matrix theory. We employed the Benjamini and Hochberg false discovery rate to adjust correlation *P* values (*P* < 0.05) (65). There are many algorithms and approaches for constructing co-occurrence networks: ensemble approach, sparse correlation, local similarity analysis, or maximal information coefficient, each with advantages and disadvantages (66). The RMT-based method has four strengths: (i) it has a solid theory base since it is built on the two universal laws of RMT; (ii) its automatic threshold calculation eliminates artificial threshold setting, which is a common and severe issue and a primary source of uncertainty in co-occurrence network creation; (iii) it effectively eliminates noise generated by system-specific properties and nonrandom; and (iv) it supports ecological data sets of various formats (12, 54).

We generated sub-networks for each soil sample using the igraph package’s subgraph function following Ma et al. (67). To determine network complexity, we calculated topographical features, including average degree, node and edge numbers, connectance, and clustering coefficient for each subnetwork. Higher above topographical feature values indicate higher network complexity (33, 68). Furthermore, the absolute negative to positive cohesion ratio (|negative|:positive cohesion) and network vulnerability were used to assess network stability (12, 69). In general, networks with higher |negative|:positive cohesion and lower network vulnerability tend to be more stable. Detailed instructions on how to determine network vulnerability are available in the supplemental material.

Ecosystems provide a variety of services and functions simultaneously, instead of a single measurable process (70). Considering that nutrient cycling is the key soil ecosystem process that supports human well-being (71), we calculated the multinutrient cycling index to assess soil ecosystem functioning (53, 70). Detailed information for quantifying the multinutrient cycling index is provided in the supplemental material.

Linear mixed-effects models were applied to assess the effects of experimental treatment on soil multinutrient cycling, microfauna network complexity and stability, and microfauna functional groups (7). In these models, individual warming treatment was treated as a fixed effect, whereas blocks were treated as random intercept effects. We used regression coefficients to assess the magnitude and direction of treatment effects, i.e., effect size (β) (7).

Associations between environmental variables and microfauna network complexity and stability were assessed using linear and polynomial regression. We assessed goodness-of-fit for candidate linear and polynomial regressions using the Akaike information criterion (AIC). We evaluated network complexity using network connectance, which is strongly correlated with other network complexity indicators, such as clustering coefficient, average degree, node number, and edge number (Pearson’s *R* > 0.92, *P* < 0.001 for nematodes; Pearson’s *R* > 0.94, *P* < 0.001 for protists; Pearson’s *R* > 0.95, *P* < 0.001 for whole microfauna). We used |negative|:positive cohesion as a proxy for network stability. In addition, we assessed the influence of environmental factors on network complexity and stability using linear mixed-effect models. Hierarchical variance partitioning was applied to assess the influence of fixed effects (soil properties) on microfauna network complexity and stability, with blocks used as random effects. The analysis was done by the glmm.hp package, and we ranked the relative significance of environmental factors on soil microfauna network complexity and stability (72).

Generalized linear mixed-effects models were applied to evaluate the influence of microfauna network complexity and stability and microfauna functional groups on multinutrient cycling. Multinutrient cycling was used as the response variable, with network complexity and stability and microfauna functional groups used as fixed variables. Blocks were used as random variables. The best linear and nonlinear models were identified as those with the lowest AIC values with a difference >2 from the model with the second-best fit.

## Data Availability

Raw sequence data were submitted to NCBI with the Sequence Read Archive (BioProject accession number: PRJNA1148023).

## SUPPLEMENTAL MATERIAL

Supplemental material mbio.00156-25-s0001.docx Additional experimental details and supplemental figures.

ASM does not own the copyrights to Supplemental Material that may be linked to, or accessed through, an article. The authors have granted ASM a non-exclusive, world-wide license to publish the Supplemental Material files. Please contact the corresponding author directly for reuse.
