# Supplementary material for: Nonlinear response of soil microfauna network complexity and stability to multilevel warming in an old-growth subtropical forest
Source: mBio. 2025 Aug 29;16(10):e00156-25. doi: 10.1128/mbio.00156-25 (PMC12505885; doi:10.1128/mbio.00156-25)
Supplement: Supplemental material — Additional experimental details and supplemental figures. [file mbio.00156-25-s0001.docx]

**Supplementary Material for:**

**Nonlinear response of soil microfauna network complexity and stability to multilevel warming in an old-growth subtropical forest**

**Authors:** Debao Li^1,2^, Yan Li^1,2^, Haibian Xu^1,2^, Jianping Wu^1,2*^

**Affiliations:** ^1^Ministry of Education Key Laboratory for Transboundary Ecosecurity of Southwest China, Yunnan Key Laboratory of Plant Reproductive Adaptation and Evolutionary Ecology and Institute of Biodiversity, School of Ecology and Environmental Science, Yunnan University, Kunming 650500, China

^2^Laboratory of Soil Ecology and Health in Universities of Yunnan Province, Yunnan University, Kunming 650500, China

***Correspondence:** Prof. Jianping Wu, E-mail: jianping.wu@ynu.edu.cn; Tel: +86-871-65939546

**MATERIALA AND METHODS**

**Experimental design**

The heating is carried out using infrared radiator. In warming plots, three 120 cm lamps were arranged in a triangle formation, positioned 1 m above the soil surface, and continuously powered for heating. The lamps were angled at 30° to the ground to optimize heat gathering. Since May 2019, the heating is on 24 hours per day and 365 days per year in the field. Infrared radiator with different wattages were used to achieve different warming levels: no warming (control), three 100W lamps to increase temperature (0.8 ℃ warming), three 200W lamps to increase temperature (1.5 ℃ warming), three 400W lamps to increase temperature (3.0 ℃ warming), and three 600W lamps to increase temperature (4.2 ℃ warming). To quantify the influence of infrared radiator on soil environmental factors, soil temperature and moisture recorders (Shengyan Electronic Technology Co.Ltd., Handan, Heibei, China) were used in each plot to measure soil temperature and moisture at soil surface, and the data were recorded every 20 min. The warmed temperature was the actual value we measured from May 2019 to August 2022. The distance between each two adjacent plots is at least 3 m from sides to avoid heating of the control plots. The temperature treatments were controlled by sensors and the heating method has been widely used in previous studies (1-4).

**Soil physicochemical characteristics measurements**

Soil pH was measured using PHS-3C (Shanghai Leici) after shaking in soil-water (1:2.5 w/v) suspension for 30 min. Soil moisture content was determined using 10 g of fresh soil dried at 105 ℃ for 48 h to constant weight. Soil organic carbon of air-dried soils were measured with an elemental analyzer after being ground by a ball mill (MAT-253; Thermo Fisher Scientific). Hourly data on soil temperature at 5 cm depth in control and warming plots were automatically collected through LI-8150-203 soil temperature probes. The soil microbial biomass carbon, microbial biomass nitrogen, and microbial biomass phosphorous contents were determined by the chloroform fumigation-extraction method (5-7). Soil total nitrogen was determined using the Kjeldahl technique. Soil total phosphorous was evaluated via a colorimetric technique after digestion with sulfuric and perchloric acids. Soil NH_4_^+^-N and NO_3_^-^-N were extracted using 2 mol L^-1^ KCL, and their concentrations were measured with a continuous flow analyzer (AutoAnalyzer-AA3, Seal Analytica, Germany). Soil available phosphorous was determined using the Olsen technique. Soil dissolved organic carbon was extracted using 0.05 M potassium sulfate and determined using the dichromate oxidation method (8).

**DNA extraction, amplification and sequencing**

Total genomic DNA was extracted from 0.5 g of fresh soil using the Powersoil DNA Isolation Kit (MoBio Laboratories, Carlsbad, CA, USA) following the manufacturer's instructions. The extracted DNA was quantified and qualified using a Nanodrop ND-1000 spectrophotometer at 260/280 and 260/230 ratios ≥1.8 (NanoDrop Technologies, Delaware, USA). The DNA was then used for high-throughput sequencing of the nematodes and protists community.

The protist and nematode 18S rRNA genes were amplified using primer pairs TAReuk454FWD1/TAReukREV3 (9) and NF1-F/18Sr2b-R (10). The taxonomic profiles of soil protist and nematode communities were determined via amplicon sequencing using the Illumina MiSeq platform.

**Bioinformatics analyses**

Bioinformatics analyses were conducted using QIIME2. Raw sequence data was demultiplexed by the demux plugin, after which primers were cut by the cutadapt plugin. Sequences were then quality filtered and denoised using the DADA2 plugin. Purified paired-end sequences were combined to produce amplicon sequence variants (ASVs) at the 100% identity level. Representative sequences were assigned to taxonomic lineages using the PR2 database for protists and the SILVA database (release 138) for nematodes. Nematode functional groups (predators, omnivores, herbivores, fungivores, and bacterivores) were determined using the nematode physiological parameter database (<http://nemaplex.ucdavis.edu>). We assigned protist ASVs to three main functional groups: phototrophs, parasites, and consumers after Mazel et al. (11).

**Network vulnerability**

Vulnerability refers to how quickly the consequence of biological/ecological events traverse to parts or the entire network, and is characterized by the maximal vulnerability of nodes in the network (12):

$$Vulnerability=max(\frac{E-E_{i}}{E})$$

where *E* is the global efficiency and *E_i_* is the global efficiency after removing node *i* and its entire links (13). The global efficiency of a graph was calculated as the average of the efficiencies over all pairs of nodes (12):

$$E=\frac{1}{n(n-1)}\sum_{i\neq j} \frac{1}{d(i,j)}$$

where *d(i,j)* is the number of edges in the shortest path between node *i* and *j*. Efficiency describes how fast information spread within the network (12).

**Multinutrient cycling index**

This index comprised information for seven soil nutrient variables in relation to carbon (soil organic carbon and dissolved organic carbon), nitrogen (total nitrogen, NH_4_^+^-N, and NO_3_^-^-N), and phosphorus (total phosphorus and available phosphorus) cycling. These variables constitute an integrated proxy for nutrient cycling and are important determinants of ecosystem functioning in terrestrial ecosystems (14-16). To derive a quantitative multinutriemt cycling index value for each sample, we averaged the standardized scores (a common scale ranging from 0 to 1) of all individual nutrient variables, as described by Jiao et al. (14). This method was used to quantify soil multinutrient cycling because it is a straightforward and interpretable measure of a community’s ability to sustain multiple functions simultaneously (14-16).

**Statistical analyses**

All statistical analyses were completed in the R software (v4.2.2; <https://www.r-project.org/>), using “vegan” (17), “ggplot2” (18), “car” (19), “multcomp” (20), “psych” (21), “reshape2” (22), “MASS” (23), “MuMIn” (24), “phyloseq” (25), “picante” (26), “stats” (27), “ecodist” (28), “igraph” (29), “lme4” (30), “nlme” (31), “glmm.hp” (32), “glmmTMB” (33), “WGCNA” (34), and “ape” (35) packages.

**REFERENCES**

1. Quan Q, Tian D, Luo Y, Zhang F, Crowther TW, Zhu K, Chen HYH, Zhou Q, Niu S. 2019. Water scaling of ecosystem carbon cycle feedback to climate warming. Sci Adv 5:eaav1131.

2. Wu L, Zhang Y, Guo X, Ning D, Zhou X, Feng J, Yuan MM, Liu S, Guo J, Gao Z, Ma J, Kuang J, Jian S, Han S, Yang Z, Ouyang Y, Fu Y, Xiao N, Liu X, Wu L, Zhou A, Yang Y, Tiedje JM, Zhou J. 2022. Reduction of microbial diversity in grassland soil is driven by long-term climate warming. Nat Microbiol 7:1054-1062.

3. Zhang Y, Ning D, Wu L, Yuan MM, Zhou X, Guo X, Hu Y, Jian S, Yang Z, Han S, Feng J, Kuang J, Cornell CR, Bates CT, Fan Y, Michael JP, Ouyang Y, Guo J, Gao Z, Shi Z, Xiao N, Fu Y, Zhou A, Wu L, Liu X, Yang Y, Tiedje JM, Zhou J. 2023. Experimental warming leads to convergent succession of grassland archaeal community. Nat Clim Chang 13:561-569.

4. Tao X, Yang Z, Feng J, Jian S, Yang Y, Bates CT, Wang G, Guo X, Ning D, Kempher ML, Liu XJA, Ouyang Y, Han S, Wu L, Zeng Y, Kuang J, Zhang Y, Zhou X, Shi Z, Qin W, Wang J, Firestone MK, Tiedje JM, Zhou J. 2024. Experimental warming accelerates positive soil priming in a temperate grassland ecosystem. Nat Commun 15:1178.

5. Brookes PC, Powlson DS, Jenkinson DS. 1984. Phosphorus in the soil microbial biomass. Soil Biol Biochem 16:169-175.

6. Brookes PC, Landman A, Pruden G, Jenkinson DS. 1985. Chloroform fumigation and the release of soil nitrogen: a rapid direct extraction method to measure microbial biomass nitrogen in soil. Soil Biol Biochem 17:837-842.

7. Vance ED, Brookes PC, Jenkinson DS. 1987. An extraction method for measuring soil microbial biomass C. Soil Biol Biochem 19:703-707.

8. Vance ED, Brookes PC, Jenkinson DS. 1987. Microbial biomass measurements in forest soils: the use of the chloroform fumigation-incubation method in strongly acid soils. Soil Biol Biochem 19:697-702.

9. Stoeck T, Bass D, Nebel M, Christen R, Jones MDM, Breiner H-W, Richards TA. 2010. Multiple marker parallel tag environmental DNA sequencing reveals a highly complex eukaryotic community in marine anoxic water. Mol Ecol 19:21-31.

10. Porazinska DL, Giblin-Davis RM, Fallr L, Farmerie W, Kanzaki N, Morris K, Powers TO, Tucker AE, Sung W, Thomas WK. 2009. Evaluating high-throughput sequencing as a method for metagenomic analysis of nematode diversity. Mol Ecol Resour 9:1439-1450.

11. Mazel F, Malard L, Niculita-Hirzel H, Yashiro E, Mod HK, Mitchell EAD, Singer D, Buri A, Pinto E, Guex N, Lara E, Guisan A. 2022. Soil protist function varies with elevation in the Swiss Alps. Environ Microbiol 24:1689-1702.

12. Yuan MM, Guo X, Wu L, Zhang Y, Xiao N, Ning D, Shi Z, Zhou X, Wu L, Yang Y, Tiedje JM, Zhou J. 2021. Climate warming enhances microbial network complexity and stability. Nat Clim Chang 11:343-348.

13. Deng Y, Jiang Y-H, Yang Y, He Z, Luo F, Zhou J. 2012. Molecular ecological network analyses. BMC Bioinformatics 13:113.

14. Jiao S, Peng Z, Qi J, Gao J, Wei G. 2021. Linking bacterial-fungal relationships to microbial diversity and soil nutrient cycling. mSystems 6:01052-20.

15. Jiao S, Xu Y, Zhang J, Hao X, Lu Y. 2019. Core microbiota in agricultural soils and their potential associations with nutrient cycling. mSystems 4:00313-18.

16. Li D, Ma C, Yue C, Lin N, Zhu Y, Wu J. 2025. Windfarm construction alters soil multinutrient cycling by destabilizing microfauna community in a mountain ecosystem. J Environ Manage 373:123758.

17. Oksanen J, Simpson GL, Blanchet FG, Kindt R, Legendre P, Minchin PR, O'Hara RB, Solymos P, Stevens MHH, Szoecs E, Wagner H, Barbour M, Bedward M, Bolker B, Borcard D, Carvalho G, Chirico M, Caceres MD, Durand S, Evangelista HBA, FitzJohn R, Friendly M, Furneaux B, Hannigan G, Hill MO, Lahti L, McGlinn D, Ouellette M-H, Cunha ER, Smith T, Stier A, Braak CJFT, Weedon J. 2022. Vegan: community ecology package.https://cran.r-project.org/web/packages/vegan/index.html.

18. Wickham H. 2016. ggplot2: elegant graphics for data analysis. Springer, New York.

19. Fox J, Weisberg S. 2011. An {R} companion to applied regression, second ed. Sage, Thousand Oaks CA.

20. Hothorn T, Bretz F, Westfall P. 2008. Simultaneous inference in general parametric models. Biom J 50:346-363.

21. Revelle W. 2022. Psych: procedures for psychological, psychometric, and personality research.https://cran.r-project.org/web/packages/psych/index.html.

22. Wickham H. 2007. Reshaping data with the reshape package. J Stat Softw 21:1-20.

23. Venables WN, Ripley BD. 2002. Modern Applied Statistics with S, 4th edn. Springer, New York.

24. Barton K. 2023. MuMIn: multi-model inference. R Package Version 1475https://cranr-projectorg/web/packages/MuMIn/indexhtml.

25. McMurdie PJ, Holmes S. 2013. phyloseq: an R package for reproducible interactive analysis and graphics of microbiome census data. PLoS One 8:e61217.

26. Kembel SW, Cowan PD, Helmus MR, Cornwell WK, Morlon H, Ackerly DD, Blomberg SP, Webb CO. 2010. Picante: R tools for integrating phylogenies and ecology. Bioinformatics 26:1463-1464.

27. Field A, Miles J, Field Z. 2012. Discovering statistics using R. Sage publications, London.

28. Goslee S, Urban D. 2022. Ecodist: dissimilarity-based functions for ecological analysis. R package version 209:https://cran.r-project.org/web/packages/ecodist/index.html.

29. Csardi G, Nepusz T. 2006. The igraph software package for complex network research. InterJournal, Complex Systems 1695:1-9.

30. Bates D, Mächler M, Bolker B, Walker S. 2015. Fitting linear mixed-effects models using lme4. J Stat Softw 67:1-48.

31. Pinheiro J, Bates D, DebRoy S, Sarkar D, R Core Team A. 2017. Nlme: linear and nonlinear mixed effects models.https://cran.r-project.org/web/packages/nlme/index.html.

32. Lai J, Zou Y, Zhang S, Zhang X, Mao L. 2022. glmm.hp: an R package for computing individual effect of predictors in generalized linear mixed models. J Plant Ecol 15:1302-1307.

33. Brooks ME, Kristensen K, van Benthem KJ, Magnusson A, Berg CW, Nielsen A, Skaug HJ, Machler M, Bolker BM. 2017. glmmTMB balances speed and flexibility among packages for zero-inflated generalized linear mixed modeling. R J 9:378-400.

34. Langfelder P, Horvath S. 2012. Fast R functions for robust correlations and hierarchical clustering. J Stat Softw 46:i11-i37.

35. Paradis E, Claude J, Strimmer K. 2004. APE: analyses of phylogenetics and evolution in R language. Bioinformatics 20:289-290.

**Supplementary Figures**

**
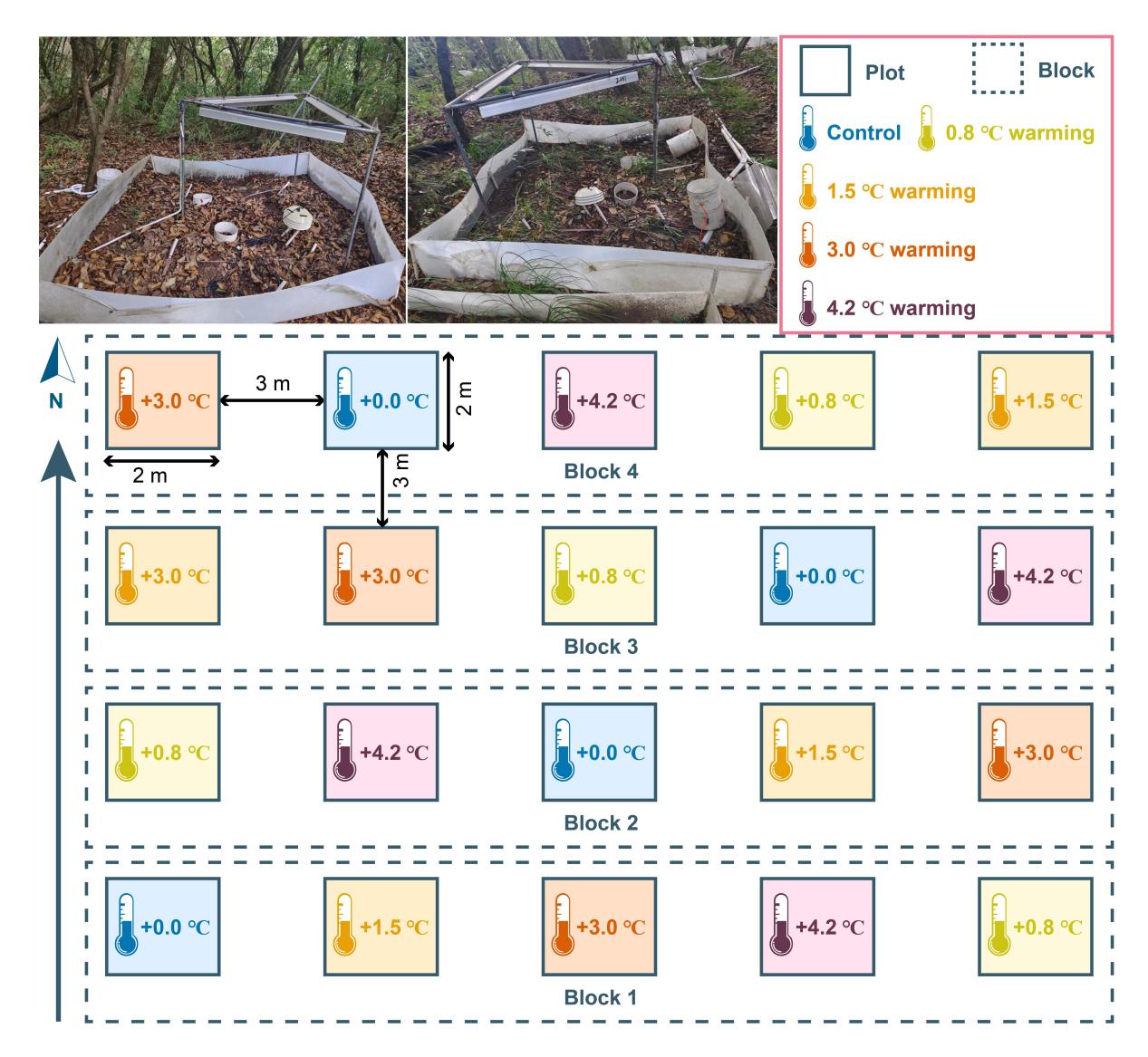
**

**Figure S1.** Experimental settings for treatments and a schematic map of the field experimental treatments.


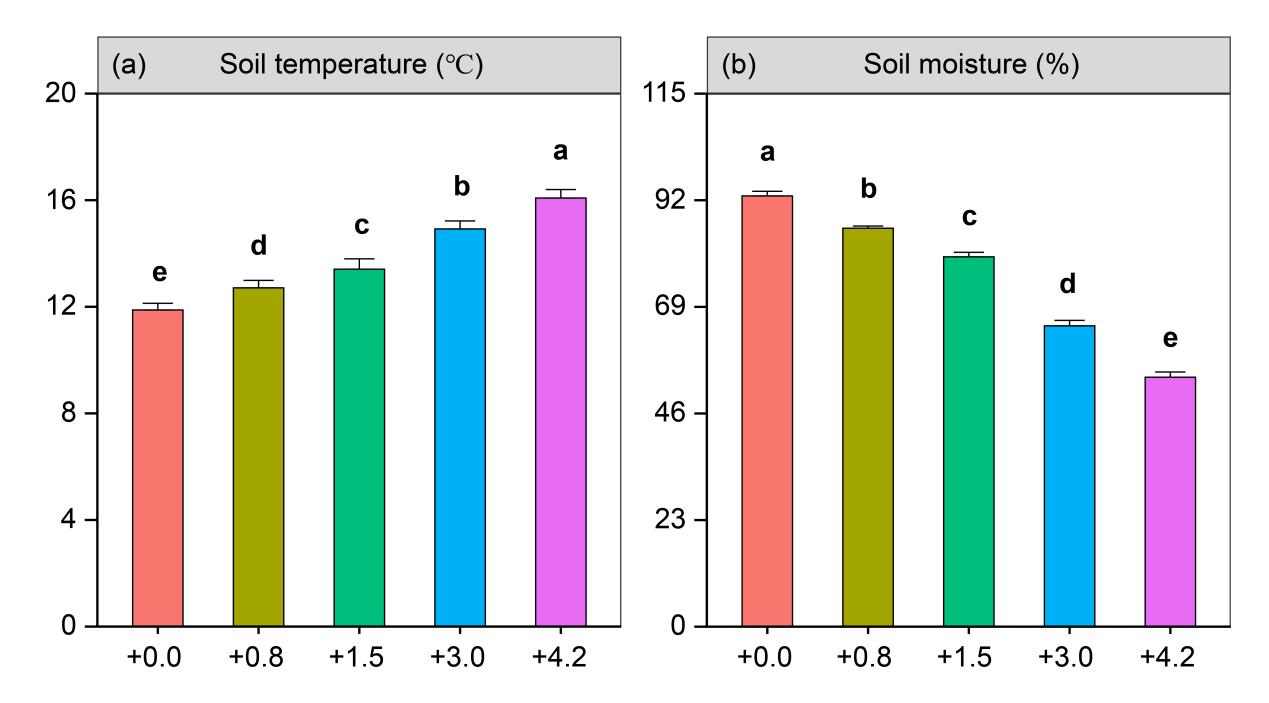


**Figure S2.** Effects of warming on soil temperature (a) and moisture (b). Distinct lowercase letters mean significant differences at *P* < 0.05 level among treatments. +0.0, control; +0.8, 0.8 ℃ above ambient temperature; +1.5, 1.5 ℃ above ambient temperature; +3.0, 3.0 ℃ above ambient temperature; +4.2, 4.2 ℃ above ambient temperature.


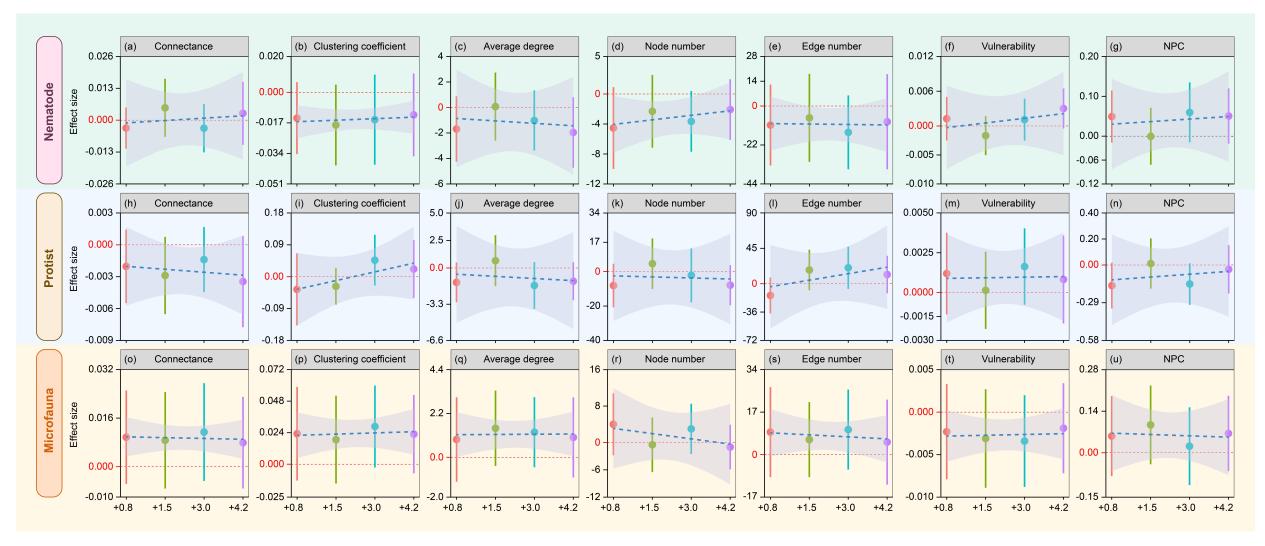


**Figure S3.** Effect sizes of multilevel warming on soil nematode (a-g), protist (h-n), and whole microfauna (o-u) network complexity and stability indices in the 10-20 cm soil layer as obtained from linear mixed-effects models. Results are expressed as mean ± standard error of the estimated effect sizes. Statistical significance is based on Wald type II χ² tests; ****P*<0.001, ***P*<0.01, **P*<0.05. Regression lines are blue and gray shading denotes 95% confidence intervals. +0.8, 0.8 ℃ above ambient temperature; +1.5, 1.5 ℃ above ambient temperature; +3.0, 3.0 ℃ above ambient temperature; +4.2, 4.2 ℃ above ambient temperature; NPC, |negative|:positive cohesion.


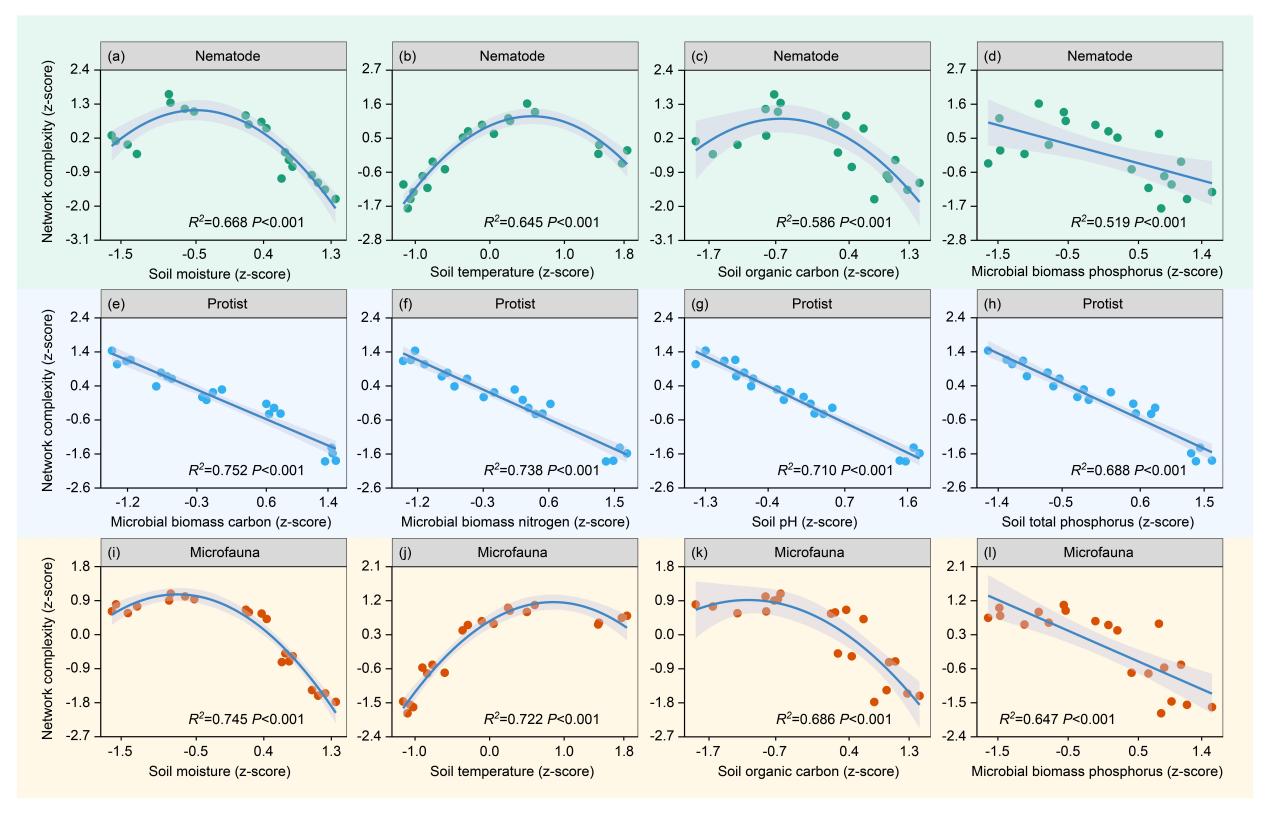


**Figure S4.** Links between soil nematode network complexity and soil moisture (a), temperature (b), organic carbon (c), and microbial biomass phosphorus (d) in the 0-10 cm soil layer. Links between soil protist network complexity and soil microbial biomass carbon (e), microbial biomass nitrogen (f), pH (g), and total phosphorus (h) in the 0-10 cm soil layer. Links between soil whole microfauna network complexity and soil moisture (i), temperature (j), organic carbon (k), and microbial biomass phosphorus (l) in the 0-10 cm soil layer. Regression lines are blue and gray shading denotes 95% confidence intervals.


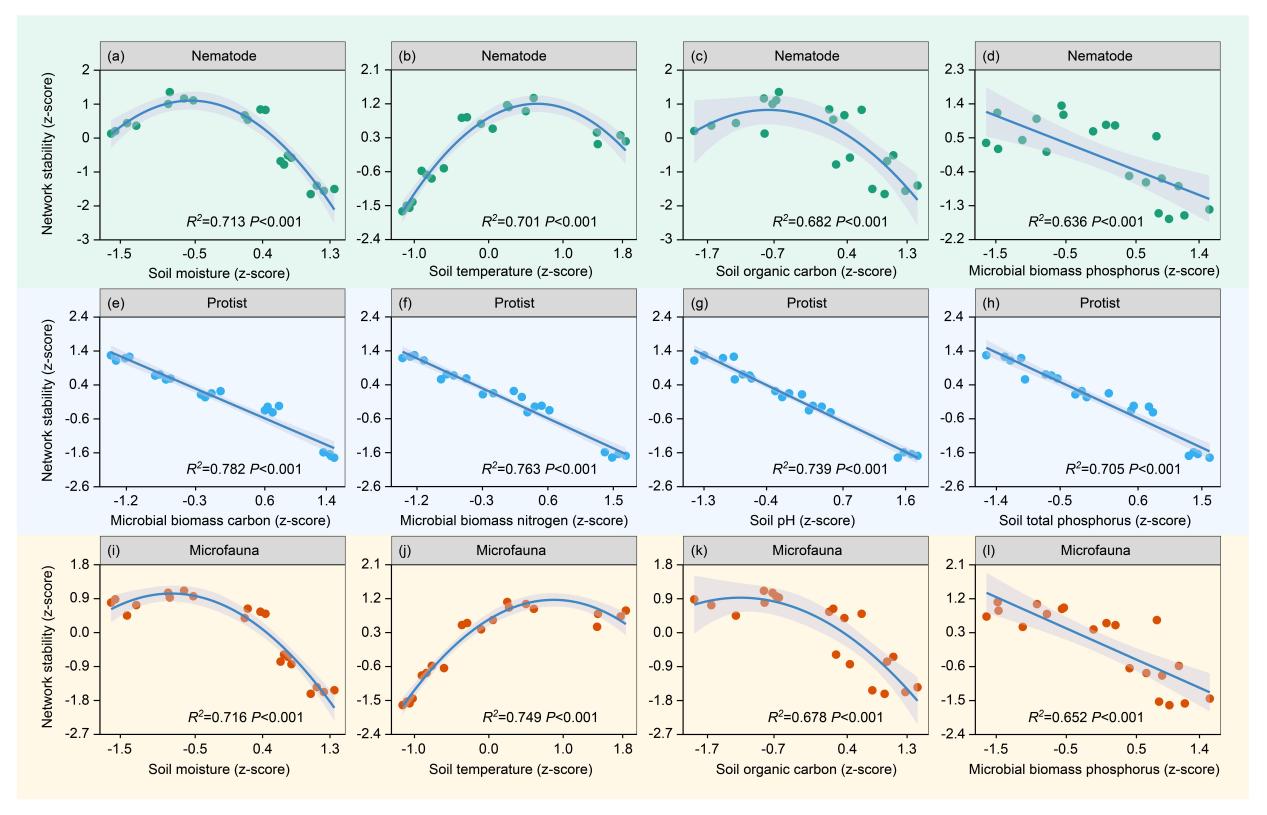


**Figure S5.** Links between soil nematode network stability and soil moisture (a), temperature (b), organic carbon (c), and microbial biomass phosphorus (d) in the 0-10 cm soil layer. Links between soil protist network stability and soil microbial biomass carbon (e), microbial biomass nitrogen (f), pH (g), and total phosphorus (h) in the 0-10 cm soil layer. Links between soil whole microfauna network stability and soil moisture (i), temperature (j), organic carbon (k), and microbial biomass phosphorus (l) in the 0-10 cm soil layer. Regression lines are blue and gray shading denotes 95% confidence intervals.


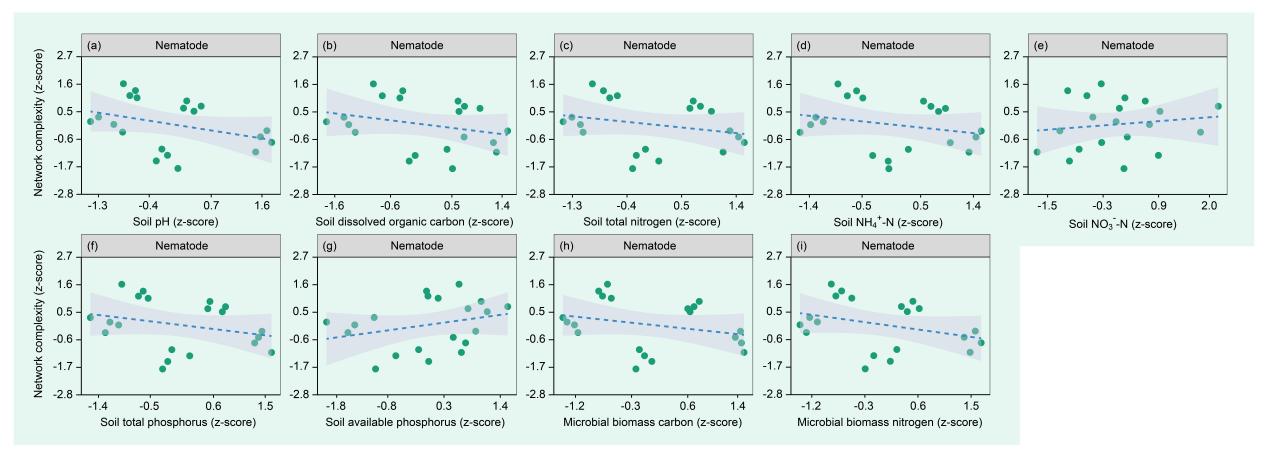


**Figure S6.** Links between soil nematode network complexity and soil pH (a), dissolved organic carbon (b), total nitrogen (c), NH_4_^+^-N(d), NO_3_^-^-N (e), total phosphorus (f), available phosphorus (g), microbial biomass carbon (h), and microbial biomass nitrogen (i) in the 0-10 cm soil layer. Regression lines are blue and gray shading denotes 95% confidence intervals.


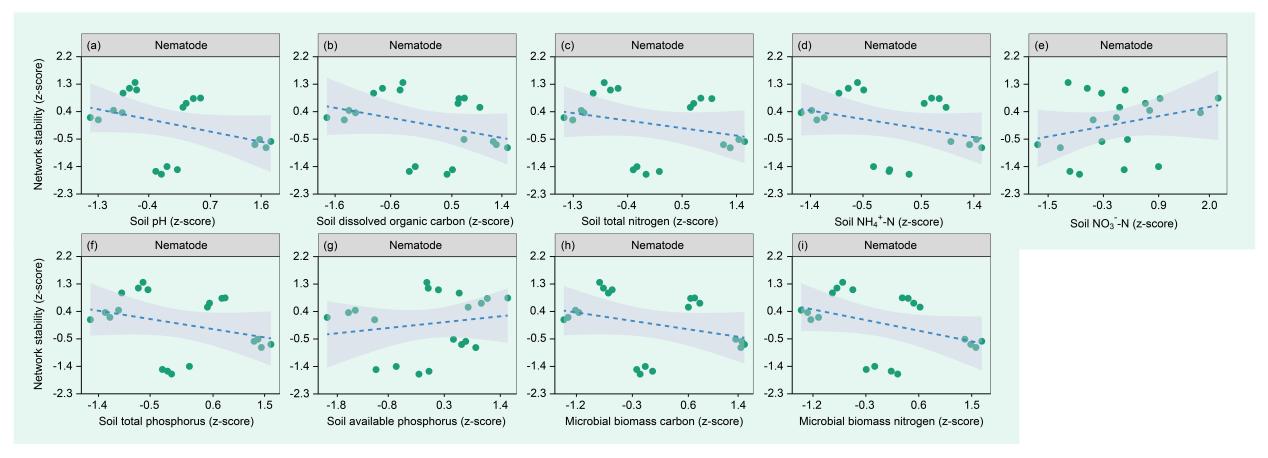


**Figure S7.** Links between soil nematode network stability and soil pH (a), dissolved organic carbon (b), total nitrogen (c), NH_4_^+^-N(d), NO_3_^-^-N (e), total phosphorus (f), available phosphorus (g), microbial biomass carbon (h), and microbial biomass nitrogen (i) in the 0-10 cm soil layer. Regression lines are blue and gray shading denotes 95% confidence intervals.


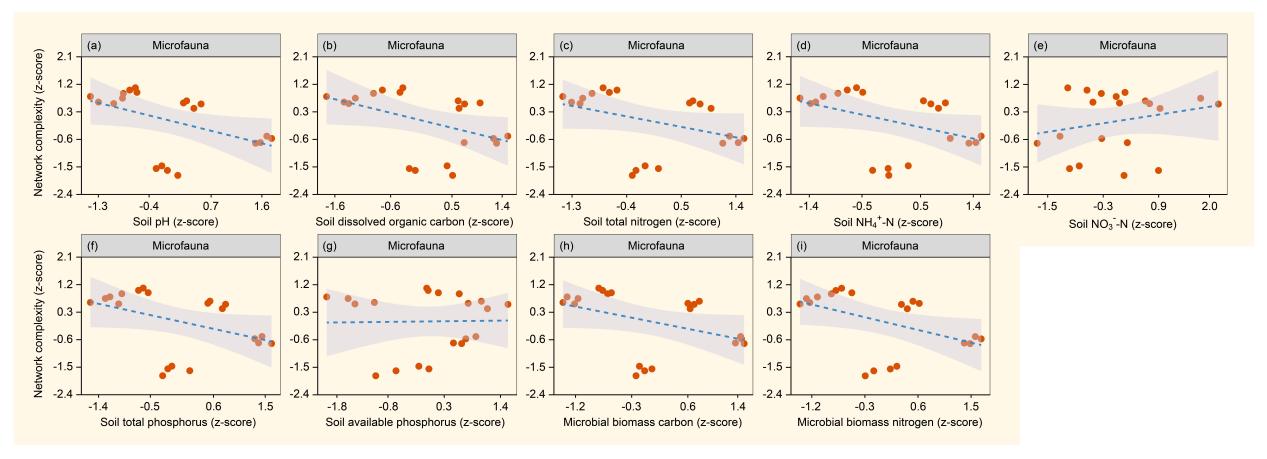


**Figure S8.** Links between soil whole microfauna network complexity and soil pH (a), dissolved organic carbon (b), total nitrogen (c), NH_4_^+^-N(d), NO_3_^-^-N (e), total phosphorus (f), available phosphorus (g), microbial biomass carbon (h), and microbial biomass nitrogen (i) in the 0-10 cm soil layer. Regression lines are blue and gray shading denotes 95% confidence intervals.


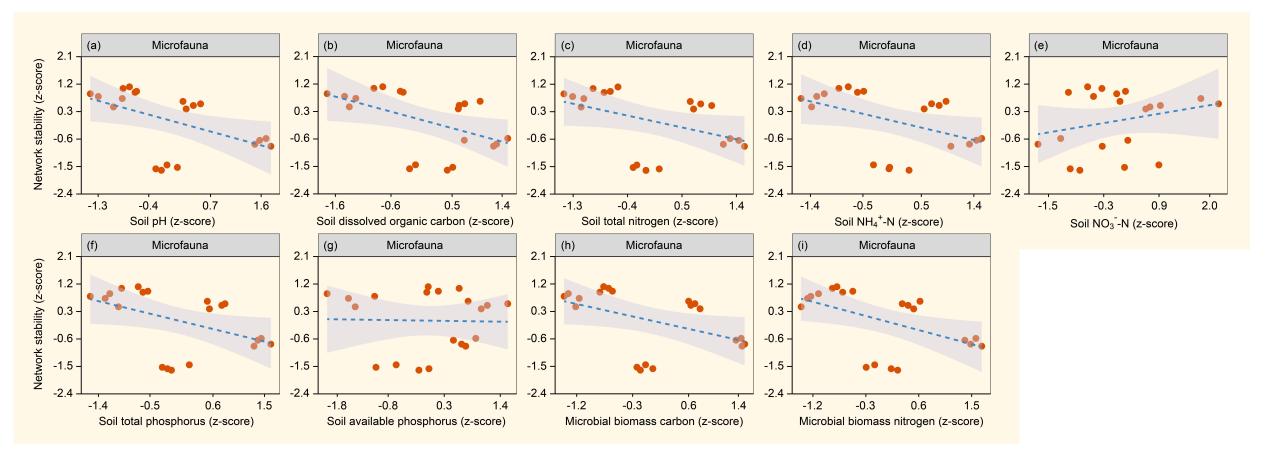


**Figure S9.** Links between soil whole microfauna network stability and soil pH (a), dissolved organic carbon (b), total nitrogen (c), NH_4_^+^-N(d), NO_3_^-^-N (e), total phosphorus (f), available phosphorus (g), microbial biomass carbon (h), and microbial biomass nitrogen (i) in the 0-10 cm soil layer. Regression lines are blue and gray shading denotes 95% confidence intervals.


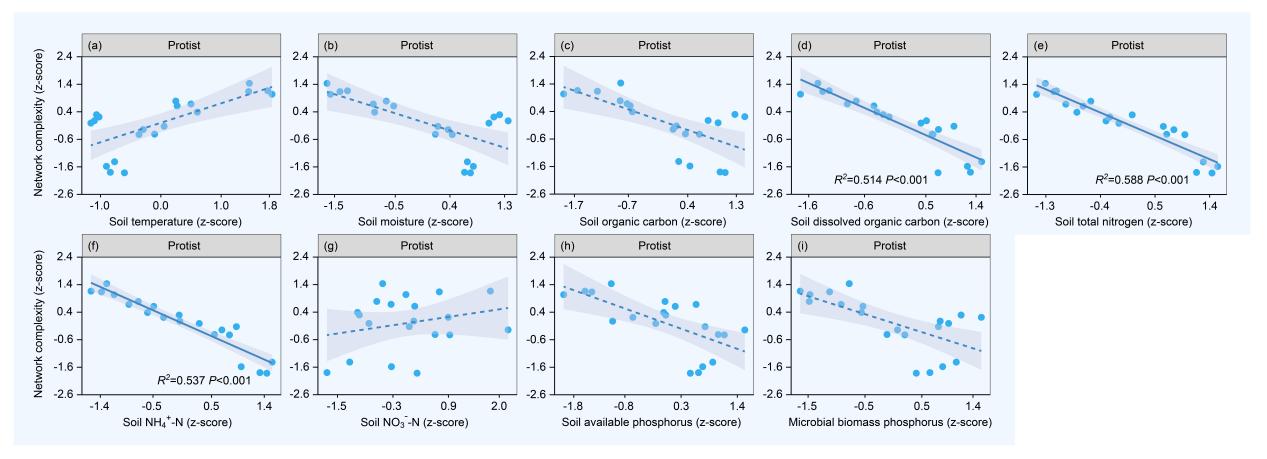


**Figure S10.** Links between soil protist network complexity and soil temperature (a), moisture (b), organic carbon (c), dissolved organic carbon (d), total nitrogen (e), NH_4_^+^-N (f), NO_3_^-^-N (g), available phosphorus (h), and microbial biomass phosphorus (i) in the 0-10 cm soil layer. Regression lines are blue and gray shading denotes 95% confidence intervals.


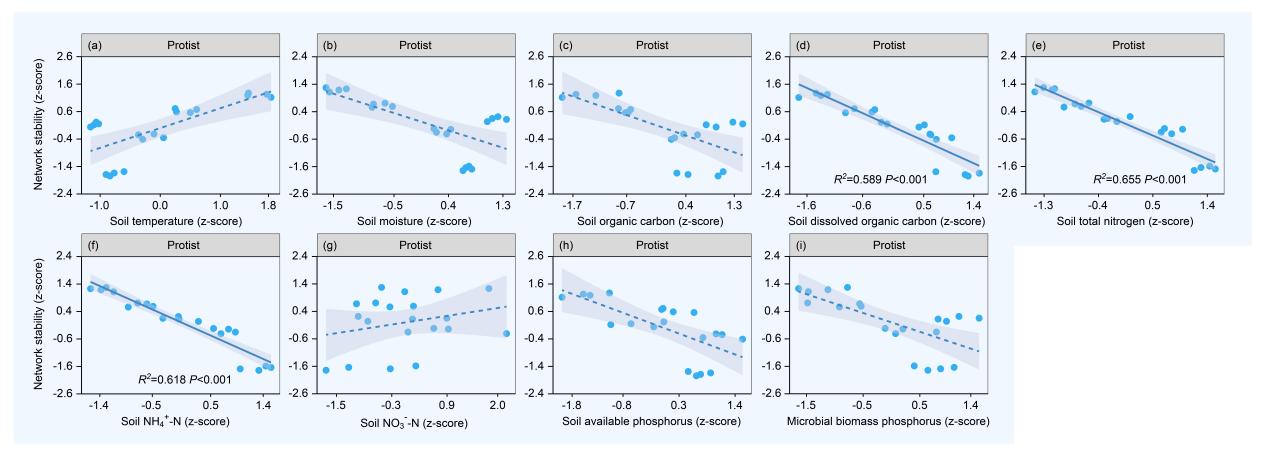


**Figure S11.** Links between soil protist network stability and soil temperature (a), moisture (b), organic carbon (c), dissolved organic carbon (d), total nitrogen (e), NH_4_^+^-N (f), NO_3_^-^-N (g), available phosphorus (h), and microbial biomass phosphorus (i) in the 0-10 cm soil layer. Regression lines are blue and gray shading denotes 95% confidence intervals.


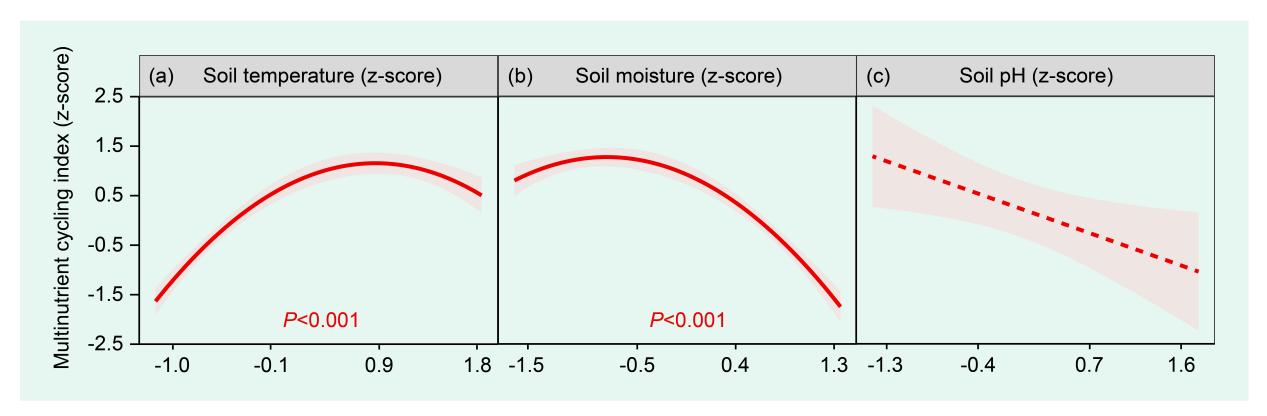


**Figure S12.** Links between soil temperature (a), moisture (b), and pH (c) and multinutrient cycling in the 0-10 cm soil layer, as obtained from generalized linear mixed-effects models. Lines and shaded polygons indicate generalized linear mixed-effects models predictions and their 95% confidence intervals.


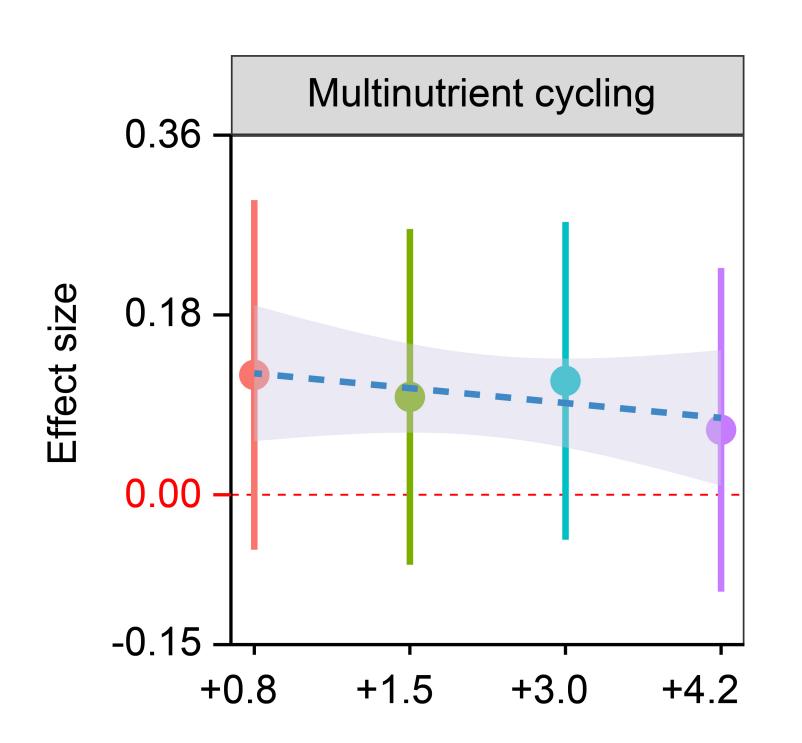


**Figure S13.** Effect sizes of multilevel warming on multinutrient cycling in the 10-20 cm soil layer as obtained from linear mixed-effects models. Results are expressed as mean ± standard error of the estimated effect sizes. Statistical significance is based on Wald type II χ² tests; ****P*<0.001, ***P*<0.01, **P*<0.05. Regression lines are blue and gray shading denotes 95% confidence intervals.


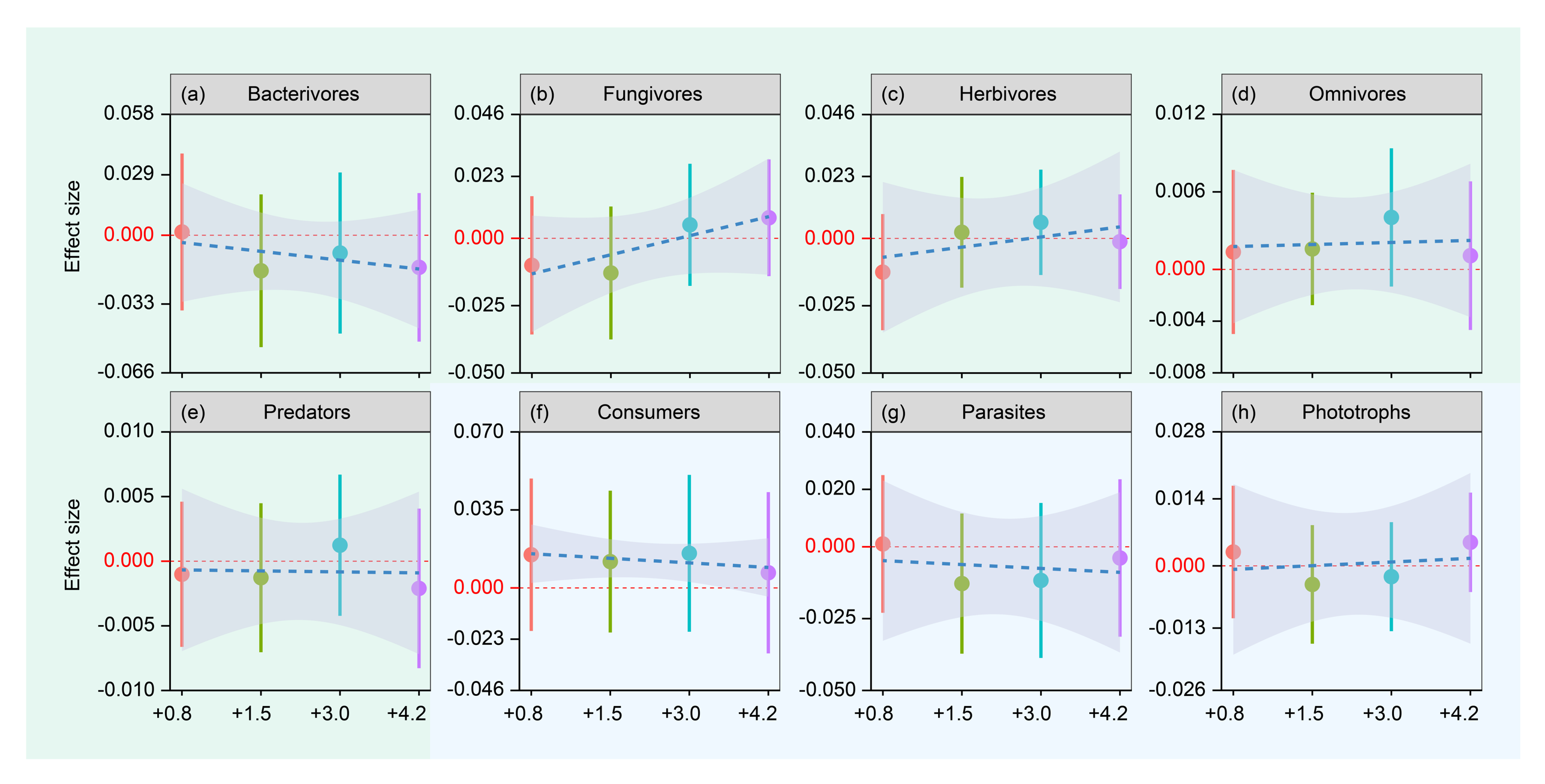


**Figure S14.** Effect sizes of multilevel warming on the relative abundance of nematode functional groups (a-e) and protist functional groups (f-h) in the 10-20 cm soil layer as obtained from linear mixed-effects models. Results are expressed as mean ± standard error of the estimated effect sizes. Statistical significance is based on Wald type II χ² tests; ****P*<0.001, ***P*<0.01, **P*<0.05. Regression lines are blue and gray shading denotes 95% confidence intervals. +0.8, 0.8 ℃ above ambient temperature; +1.5, 1.5 ℃ above ambient temperature; +3.0, 3.0 ℃ above ambient temperature; +4.2, 4.2 ℃ above ambient temperature.
